# Real-world efficacy, safety, and treatment continuity of fruquintinib in Japanese patients with colorectal cancer: a multicentre retrospective study

**DOI:** 10.1016/j.esmogo.2026.100418

**Published:** 2026-09-22

**Authors:** T. Ito, K. Shiraishi, Y. Ishizuka, N. Ishibashi, S. Kameishi, Y. Kito, A. Sugaya, N. Takahashi, H. Kodama, S. Ueno, H. Sagara, S. Kurata, F. Hanamura, K. Hino, Y. Kono, T. Yoshihiro, T. Sakakida, T. Koyama, M. Wakabayashi, N. Suzuki, T. Takayama, N. Tsuruta, K. Baba, A. Nakata, K. Hirata

**Affiliations:** 1 Department of Medical Oncology, NHO Nagoya Medical Center, Nagoya, Japan; 2 Department of Clinical Oncology, Aichi Cancer Center, Nagoya, Japan; 3 Division of Gastrointestinal Oncology, Shizuoka Cancer Center, Shizuoka, Japan; 4 Department of Gastrointestinal Medical Oncology, National Cancer Center Hospital, Tokyo, Japan; 5 Department of Medical Oncology, Ishikawa Prefectural Central Hospital, Kanazawa, Japan; 6 Department of Medical Oncology, Ibaraki Prefectural Central Hospital, Kasama, Japan; 7 Department of Gastroenterology, Saitama Cancer Center, Saitama, Japan; 8 Cancer Chemotherapy Center, Osaka Medical and Pharmaceutical University Hospital, Takatsuki, Japan; 9 Department of Hematology, Oncology and Cardiovascular Medicine, Kyushu University Hospital, Fukuoka, Japan; 10 Department of Pharmacy, Nakadori General Hospital, Akita, Japan; 11 Division of Gastroenterology and Hepatology, Department of Internal Medicine, Keio University School of Medicine, Tokyo, Japan; 12 Department of Medical Oncology, Hamanomachi Hospital, Fukuoka, Japan; 13 Department of Gastrointestinal Medical Oncology, NHO Shikoku Cancer Center, Matsuyama, Japan; 14 Department of Gastroenterology and Hepatology, Okayama University Hospital, Okayama, Japan; 15 Department of Hematology and Oncology, Japan Community Healthcare Organization Kyushu Hospital, Kitakyushu, Japan; 16 Department of Molecular Gastroenterology and Hepatology, University Hospital, Kyoto Prefectural University of Medicine, Kyoto, Japan; 17 Department of Medical Oncology and Hematology, Kobe University Hospital, Kobe, Japan; 18 Department of Gastroenterology and Hepatology, Toho University Omori Medical Center, Tokyo, Japan; 19 Department of Gastroenterology, Graduate School of Medicine, The University of Tokyo, Tokyo, Japan; 20 Department of Medical Oncology, Daido Hospital, Nagoya, Japan; 21 Tsuruta Home Care Medical Institution, Tendai Medical Corporation, Fukuoka, Japan; 22 Department of Oncology and General Medicine, IMSUT Hospital, The Institute of Medical Science Hospital, The University of Tokyo, Tokyo, Japan; 23 Department of Gastroenterology, Osaka Metropolitan University Hospital, Japan, Osaka, Japan

**Keywords:** colorectal cancer, fruquintinib, Japan, proteinuria, real-world evidence

## Abstract

**Background:**

Fruquintinib has recently been introduced as a later-line treatment option for unresectable or recurrent colorectal cancer (CRC) in Japan. However, real-world evidence from Japanese clinical practice remains limited; therefore, we evaluated the efficacy and safety of fruquintinib in Japanese patients with unresectable or recurrent CRC.

**Patients and methods:**

This multicentre retrospective study was conducted at 23 institutions in Japan. Patients who received fruquintinib monotherapy between November 2024 and July 2025 were included. The primary endpoint was real-world progression-free survival (rwPFS). The secondary endpoints included overall survival (OS), disease control rate (DCR), relative dose intensity, and safety.

**Results:**

Of the 185 enrolled patients, 171 were included in the analysis. The median rwPFS and OS periods were 2.5 and 5.8 months, respectively, and the DCR was 38.1%; these effectiveness outcomes were numerically less favourable than those reported in FRESCO-2. Liver and peritoneal metastases and hypoalbuminaemia were associated with poorer outcomes. Initial dose reduction was independently associated with shorter rwPFS but not with OS. Treatment-emergent adverse events (TEAEs) occurred in 95.9% of the patients and grade ≥3 TEAEs occurred in 49.1%. Proteinuria was the most frequent adverse event and the leading cause of treatment modification. Baseline hypertension was associated with a higher incidence of proteinuria.

**Conclusions:**

Fruquintinib demonstrated modest real-world activity with no new safety signals in Japanese clinical practice. However, proteinuria frequently led to treatment modification, highlighting the importance of careful toxicity management and sustained treatment continuity to maximise clinical benefit.

## Highlights

• Real-world effectiveness was numerically lower than in FRESCO-2.
• Initial dose reduction was associated with shorter rwPFS but not with OS.
• Proteinuria was the most common toxicity and led to treatment modification.
• Careful toxicity management may support treatment continuity.

## Introduction

Colorectal cancer (CRC) is the third most commonly diagnosed cancer and the second leading cause of cancer-related deaths worldwide.^1^ In Japan, CRC was the second most common cancer in 2024 and its incidence and mortality have continued to increase annually.^2^ Approximately 20% of patients present with metastatic disease and up to 50% of patients with initially localised disease develop metastases.^3^ Despite advances in systemic therapy, unresectable or recurrent CRC remains incurable. Therefore, treatment is aimed at prolonging survival while maintaining quality of life.^4^

Standard first- and second-line therapies consist of fluoropyrimidine-based combination regimens of oxaliplatin or irinotecan, in conjunction with molecularly targeted agents. In the later-line setting (third line and beyond), trifluridine–tipiracil with or without bevacizumab and regorafenib are established treatment options,^5^, ^6^, ^7^ each supported by evidence from phase III trials. Recently, fruquintinib, a highly selective oral inhibitor of vascular endothelial growth factor receptor (VEGFR)-1, VEGFR-2, and VEGFR-3, has emerged as an additional later-line treatment option owing to its antiangiogenic activity.^8^

In the global phase III FRESCO-2 trial involving patients with refractory, unresectable, or recurrent CRC,^9^ fruquintinib significantly improved overall survival (OS) and progression-free survival (PFS) compared with placebo. The median OS periods were 7.4 and 4.8 months in the fruquintinib and placebo groups, respectively [hazard ratio (HR) 0.66, 95% confidence interval (CI) 0.55-0.80, *P* < 0.001], whereas the median PFS periods were 3.7 and 1.8 months, respectively (HR 0.32, 95% CI 0.27-0.39, *P* < 0.001). A subgroup analysis in Japanese patients demonstrated a similar trend, with median OS periods of 6.9 and 5.6 months (HR 0.42, 95% CI 0.19-0.92, *P* = 0.055) and median PFS periods of 3.6 and 1.8 months (HR 0.27, 95% CI 0.13-0.56, *P* < 0.05), respectively.^10^

Accordingly, fruquintinib was approved in Japan in September 2024 as a new standard treatment option in the later-line setting for patients with unresectable or recurrent CRC. Regarding safety, a higher proportion of patients who received fruquintinib experienced grade ≥3 treatment-emergent adverse events (TEAEs) in the Japanese subgroup than in the overall population.^10^ Consistent findings have also been reported in the CORRECT trial of regorafenib,^11^ another oral tyrosine kinase inhibitor with antiangiogenic activity, supporting the need for careful toxicity management in Japanese patients. Given the potentially greater susceptibility of Japanese patients to VEGFR inhibitor-related toxicities and the increasingly common sequential use of antiangiogenic agents in later-line treatment, understanding the tolerability, treatment continuity, and toxicity profile of fruquintinib in routine clinical practice is clinically important.

However, the highly selected patient population and controlled conditions of phase III trials may limit the generalisability of these results to routine clinical practice. Accordingly, real-world evidence regarding fruquintinib use, which remains limited, is required to inform treatment selection and dosing decisions. Therefore, this multicentre retrospective study aimed to evaluate the effectiveness, tolerability, and treatment continuity of fruquintinib in Japanese patients treated in routine clinical practice.

## Materials and methods

### Study design and patients

This multicentre, retrospective study was conducted at 23 institutions in Japan. The eligibility criteria were (i) age ≥18 years at treatment initiation; (ii) histologically confirmed colorectal adenocarcinoma; (iii) receipt of fruquintinib monotherapy between 22 November 2024 and 31 July 2025; and (iv) evaluable disease, including nonmeasurable lesions. Patients were excluded if they did not meet the eligibility criteria or had insufficient clinical data for efficacy or safety evaluation. The data cut-off date for this analysis was 31 October 2025. Baseline data collected from medical records included demographic and clinical characteristics, prior treatment history, metastatic sites, laboratory parameters, and comorbidities, particularly hypertension and diabetes mellitus (DM). Hypertension was defined as either physician-diagnosed hypertension requiring ongoing antihypertensive treatment or repeated records of systolic blood pressure ≥160 mmHg or diastolic blood pressure ≥100 mmHg. DM was defined as treatment with oral hypoglycaemic agents or insulin.

### Endpoints and statistical analyses

The primary endpoint was real-world progression-free survival (rwPFS), defined as the time from the initiation of fruquintinib treatment to documented disease progression or death from any cause, whichever occurred first. Patients without documented disease progression were censored on the date of their last confirmed progression-free status. The secondary endpoints included OS, disease control rate (DCR), relative dose intensity (RDI), and safety. OS was defined as the time from treatment initiation to death from any cause and patients alive at the time of data cut-off were censored at the date of the last confirmed survival. The tumour response was assessed according to the RECIST version 1.1 by treating investigators at each institution.^12^ Radiological assessments were carried out according to routine clinical practice at each institution. DCR was defined as the proportion of patients who achieved complete response (CR), partial response (PR), or stable disease (SD) as the best overall response. The RDI was calculated as 100% × (actual dose intensity/planned dose intensity), where the planned dose intensity was set to 5 mg/day for 21 days in a 28-day cycle. The 12-week RDI was calculated from treatment initiation to week 12 or until treatment discontinuation if treatment was discontinued earlier; a separate analysis was carried out among patients who remained on treatment for at least 12 weeks. Adverse events (AEs) were evaluated by each investigator and graded according to the Common Terminology Criteria for Adverse Events version 5.0.^13^ The rwPFS and OS were estimated using the Kaplan–Meier method and the median survival times with 95% CIs were reported. Subgroup analyses of rwPFS and OS were carried out using univariate Cox proportional hazards models and HRs with 95% CIs were estimated. Multivariate Cox proportional hazards analyses were similarly carried out using variables that were significant in the univariate analyses, as well as covariates considered clinically relevant. Categorical variables were compared using the chi-square or Fisher’s exact test, as appropriate. All statistical tests were two-sided and *P* value <0.05 was considered statistically significant. Statistical analyses were carried out using EZR software (R version 4.3.1) (Saitama Medical Center, Jichi Medical University, Saitama, Japan).^14^

### Ethical approval

This study was approved by the Institutional Ethics Committee of the National Hospital Organization Nagoya Medical Center as the central review board (approval date: 5 September 2025; approval number: 2025-018). The requirement for written informed consent was waived because of the retrospective nature of this study and an opt-out approach was used in accordance with institutional guidelines.

## Results

### Patient characteristics

A total of 185 patients were enrolled; 171 of them were included in the analysis. Fourteen patients were excluded: five because of incorrect enrolment and nine because the primary tumour site was outside the colorectum (appendix, *n* = 5; small intestine, *n* = 1; anal canal, *n* = 3). The median patient age was 64 (range 29-87) years (Table 1). Eastern Cooperative Oncology Group performance status (ECOG PS) was 0, 1, and ≥2 in 33.3%, 58.5%, and 8.2% of the patients, respectively. Approximately half of the patients had hypertension and ∼10% had DM. The primary tumour site was the colon in 70.2% of the patients and the rectum in 29.8% of the patients. Overall, 60.8% of the patients received at least four prior lines of systemic anticancer therapy. Notably, 84.2% of the patients had previously received trifluridine–tipiracil plus bevacizumab, whereas 32.2% had received regorafenib. The median treatment duration was 70 (range 2-322) days (Table 2). The median RDI during the first 12 weeks was 72.5% (range 10.3-100.0). Among patients who remained on treatment for at least 12 weeks (*n* = 83), the median RDI was 73.3% (range 26.3-100.0). Fruquintinib was initiated at 5 mg/day in 78.9% (*n* = 135), 4 mg/day in 12.9% (*n* = 22), and 3 mg/day in 8.2% (*n* = 14) of the patients. The main reasons for the initial dose reduction were residual toxicities from prior therapies and poor performance status, each reported in 10 patients (27.8% of reduced-dose starters), followed by advanced age in 8 patients (22.2%); other reasons included organ dysfunction and inadequate oral intake. Overall, 52 patients (30.4%) received subsequent systemic therapy.

**Table 1:** Demographics and baseline clinical characteristics

Patient characteristics	*N* = 171
Age, years	
Median (range)	64 (29-87)
≥65	82 (48.0)
Sex	
Male	93 (54.4)
Female	78 (45.6)
Body mass index, kg/m^2^	
Median (range)	21.8 (13.6-34.9)
≥18.5	150 (87.7)
ECOG PS	
0	57 (33.3)
1	100 (58.5)
≥2	14 (8.2)
Comorbidities	
Hypertension	88 (51.5)
DM	18 (10.5)
Serum AST level, IU/l	
Median (range)	28 (12-133)
≥40	55 (32.2)
Serum ALT level, IU/l	
Median (range)	18 (5-98)
≥40	21 (12.3)
Creatinine clearance, ml/min	
Median (range)	89.3 (11.6-213.5)
<50	20 (11.7)
Baseline proteinuria	
≥2+	28 (16.4)
Serum albumin level, g/dl	
Median (range)	3.6 (2.0-4.7)
<3.0	21 (12.3)
Primary tumour site^a^	
Colon right	45 (26.3)
Colon left	75 (43.9)
Rectum	51 (29.8)
Primary tumour status	
Resected	136 (79.5)
Unresected	35 (20.5)
Histology^b^	
Differentiated	153 (89.5)
Undifferentiated	10 (5.8)
Unknown	8 (4.7)
*RAS* mutation status	
Wild type	86 (50.3)
Mutant	85 (49.7)
*BRAF*^V600E^ mutation status	
Wild type	160 (93.6)
Mutant	9 (5.3)
Missing	2 (1.2)
Microsatellite or mismatch repair status	
MSS/pMMR	162 (94.7)
MSI-H/dMMR	2 (1.2)
Missing	7 (4.1)
Number of metastatic organ sites	
1	24 (14.0)
2	53 (31.0)
≥3	94 (55.0)
Metastatic organ site	
Lung	124 (72.5)
Liver	120 (70.2)
Peritoneum	64 (37.4)
Duration from initiation of first-line chemotherapy	
≥18 months	151 (88.3)
Number of previous treatment lines	
Median (range)	4 (1-9)
≥4	104 (60.8)
Previous trifluridine–tipiracil or regorafenib	
Trifluridine–tipiracil + bevacizumab	144 (84.2)
Regorafenib	55 (32.2)
Trifluridine–tipiracil monotherapy	19 (11.1)
Both	52 (30.4)
Previous anti-VEGF agents	
Bevacizumab	159 (93.0)
Ramucirumab	21 (12.3)
Aflibercept	4 (2.3)

Values are *n* (%) unless otherwise specified.

ALT, alanine aminotransferase; AST, aspartate aminotransferase; DM, diabetes mellitus; dMMR, deficient mismatch repair; ECOG PS, Eastern Cooperative Oncology Group performance status; MSI-H, microsatellite instability-high; MSS, microsatellite stable; pMMR, proficient mismatch repair; VEGF, vascular endothelial growth factor.

a Right-sided tumours were from the caecum to the proximal two-thirds of transverse colon; left-sided tumours were from the distal one-third of transverse colon to the sigmoid colon.

b Differentiated type: papillary (pap) and tubular (tub) adenocarcinoma; undifferentiated type: poorly differentiated (por), mucinous (muc), signet-ring cell (sig), and medullary (med) carcinoma.

**Table 2:** Treatment delivery

Variable	*N* = 171
Duration of treatment, days, median (range)	70 (2-322)
Patients who continued fruquintinib for >12 weeks	83 (48.5)
RDI, median (range)	
Within first 12 weeks	72.5 (10.3-100.0)
Among 12 weeks-completion patients (*n* = 83)	73.3 (26.3-100.0)
Initial daily dose (mg)	
5	135 (78.9)
4	22 (12.9)
3	14 (8.2)
Last daily dose (mg)	
5	73 (42.7)
4	57 (33.3)
3	40 (23.4)
2	1 (0.6)
Any modification^a^	128 (74.9)
Interruption	113 (66.1)
Dose reduction	82 (48.0)
Re-escalation^b^	0 (0.0)
Escalation^c^	10 (5.8)
Reason for initial dose reduction	
Toxicity by prior therapy^d^	10 (5.8)
Poor PS	10 (5.8)
Advanced age	8 (4.7)
Organ dysfunction^e^	2 (1.2)
Inadequate oral intake	2 (1.2)
Low body weight	1 (0.6)
Patient request	1 (0.6)
Planned dose escalation	1 (0.6)
Uncertain	1 (0.6)
Subsequent systemic therapy	
Any systemic therapy	52 (30.4)
Regorafenib	13 (7.6)
Panitumumab-based therapy	13 (7.6)
Oxaliplatin-based therapy	6 (3.5)
Trifluridine–tipiracil + bevacizumab	3 (1.8)
Trifluridine–tipiracil monotherapy	0 (0.0)
Others	17 (9.9)

Values are *n* (%) unless otherwise specified.

HFSR, hand–foot skin reaction; PS, performance status; RDI, relative dose intensity.

a Any dose modifications included dose reduction, interruption, re-escalation, and escalation.

b Dose higher than the last nonzero dose.

c Dose higher than the initial dose.

d Toxicity by prior therapy included proteinuria, HFSR, fatigue, and hypertension.

e Organ dysfunction included liver and renal dysfunctions.

### Efficacy

The median follow-up period was 6.0 (95% CI 5.5-8.5) months. The median rwPFS period was 2.5 (95% CI 2.3-3.2) months and the median OS period was 5.8 (95% CI 4.9-8.9) months (Figure 1). Among patients with measurable lesions, the DCR was 38.1% (95% CI 30.7-45.9) (Table 3). The best overall response was CR in 0%, PR in 1.8%, SD in 36.3%, progressive disease in 54.2%, and not evaluable in 7.7% of the patients.

**Figure 1:**
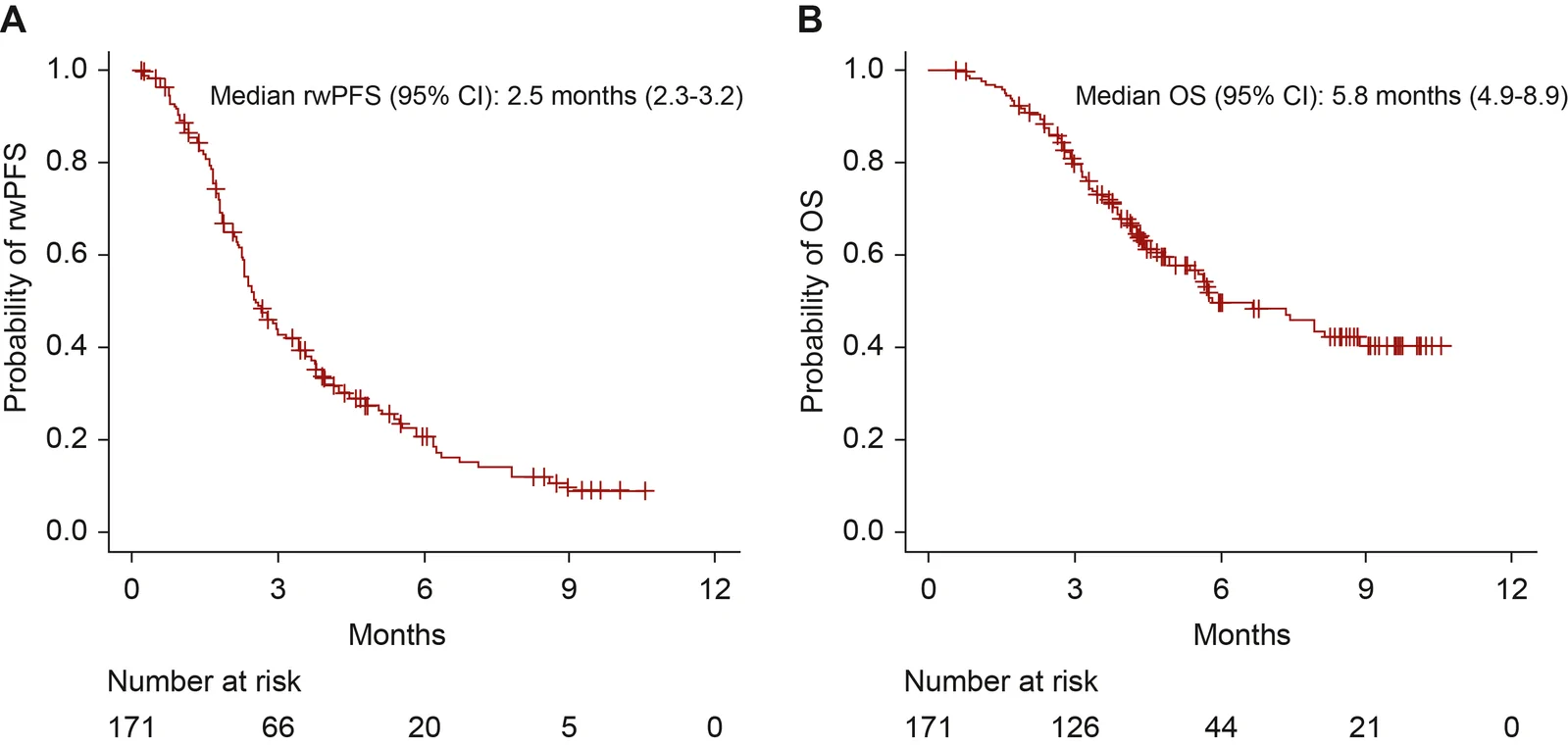
**Kaplan–Meier curves of rwPFS (A) and OS (B).** CI, confidence interval; OS, overall survival; rwPFS, real-world progression-free survival.

**Table 3:** Tumour response

Best overall response	*n* = 168	%
CR	0	0.0
PR	3	1.8
SD	61	36.3
PD	91	54.2
NE	13	7.7
ORR (95% CI)	3	1.8 (0.4-5.1)
DCR (95% CI)	64	38.1 (30.7-45.9)

Tumour response data include patients with a measurable lesion (*n* = 168).

CI, confidence interval; CR, complete response; DCR, disease control rate; NE, not evaluable; ORR, objective response rate; PD, progressive disease; PR, partial response; SD, stable disease.

### Safety

TEAEs of any grade were observed in 95.9% of the patients. Grade ≥3 TEAEs were reported in 49.1% of the patients. Serious AEs occurred in 8.8% of the patients. There were no treatment-related deaths. Proteinuria was the most frequent TEAE, occurring in 55% of the patients, including grade ≥3 events in 22.2%. Other common TEAEs included fatigue (48.5%), hand–foot skin reaction (HFSR; 43.3%), and hypertension (42.7%) (Table 4). Dose reductions and treatment interruptions owing to TEAEs occurred in 38.6% (*n* = 66) and 53.8% (*n* = 92) of the patients, respectively. Treatment was permanently discontinued owing to TEAEs in 10.5% (*n* = 18) of the patients. Proteinuria was also the most common cause of all toxicity-related treatment modifications.

**Table 4:** TEAEs

Events, *n* (%)	*N* = 171
Any grade	164 (95.9)
Grade ≥3	84 (49.1)
Serious AEs	15 (8.8)
Leading to interruption	92 (53.8)
Proteinuria	45 (26.3)
HFSR	15 (8.8)
Hypertension	14 (8.2)
Leading to dose reduction	66 (38.6)
Proteinuria	29 (17.0)
Hypertension	16 (9.4)
HFSR	12 (7.0)
Leading to discontinuation	18 (10.5)
Proteinuria	6 (3.5)
Fatigue	5 (2.9)
GI perforation	2 (1.2)
Treatment-related death	0 (0.0)

TEAEs, *n* (%)	Any grade	Grade 1	Grade 2	Grade 3	Grade 4
Proteinuria	94 (55.0)	19 (11.1)	37 (21.6)	38 (22.2)	–
Fatigue	83 (48.5)	44 (25.7)	33 (19.3)	6 (3.5)	–
HFSR	74 (43.3)	34 (19.9)	33 (19.3)	7 (4.1)	–
Hypertension	73 (42.7)	14 (8.2)	33 (19.3)	26 (15.2)	0 (0.0)
Anorexia	54 (31.6)	26 (15.2)	22 (12.9)	6 (3.5)	0 (0.0)
AST increased	51 (29.8)	35 (20.5)	8 (4.7)	7 (4.1)	1 (0.6)
Platelet count decreased	44 (25.7)	26 (15.2)	13 (7.6)	5 (2.9)	0 (0.0)
ALT increased	34 (19.9)	25 (14.6)	5 (2.9)	4 (2.3)	0 (0.0)
Anaemia	34 (19.9)	21 (12.3)	10 (5.8)	3 (1.8)	0 (0.0)
Nausea	26 (15.2)	15 (8.8)	9 (5.3)	2 (1.2)	–
Diarrhoea	24 (14.0)	10 (5.8)	11 (6.4)	3 (1.8)	0 (0.0)
Mucositis oral	21 (12.3)	12 (7.0)	8 (4.7)	1 (0.6)	0 (0.0)
Total bilirubin increased	19 (11.1)	7 (4.1)	6 (3.5)	6 (3.5)	0 (0.0)
Neutrophil count decreased	11 (6.4)	5 (2.9)	4 (2.3)	2 (1.2)	0 (0.0)
Hypothyroidism	9 (5.3)	2 (1.2)	7 (4.1)	0 (0.0)	0 (0.0)
Vomiting	4 (2.3)	3 (1.8)	1 (0.6)	0 (0.0)	0 (0.0)

AEs, adverse events; ALT, alanine aminotransferase; AST, aspartate aminotransferase; GI, gastrointestinal; HFSR, hand–foot skin reaction; TEAEs, treatment-emergent adverse events.

### Subgroup analysis

In the subgroup analysis, peritoneal metastasis, multiple metastatic sites (≥3), and hypoalbuminaemia (serum albumin level <3.0 g/dl) were associated with shorter rwPFS (Supplementary Table S1, available at https://doi.org/10.1016/j.esmogo.2026.100418). Factors associated with shorter OS included ECOG PS ≥2, liver and peritoneal metastases, multiple metastatic sites (≥3), absence of prior primary tumour resection, and hypoalbuminaemia (Supplementary Table S2, available at https://doi.org/10.1016/j.esmogo.2026.100418). A reduced starting dose was independently associated with shorter rwPFS after adjustment for these factors in the multivariate analysis (Figure 2A and Supplementary Table S1, available at https://doi.org/10.1016/j.esmogo.2026.100418), whereas it was not associated with OS in either the univariate or multivariate analysis (Figure 2B and Supplementary Table S2, available at https://doi.org/10.1016/j.esmogo.2026.100418).

**Figure 2:**
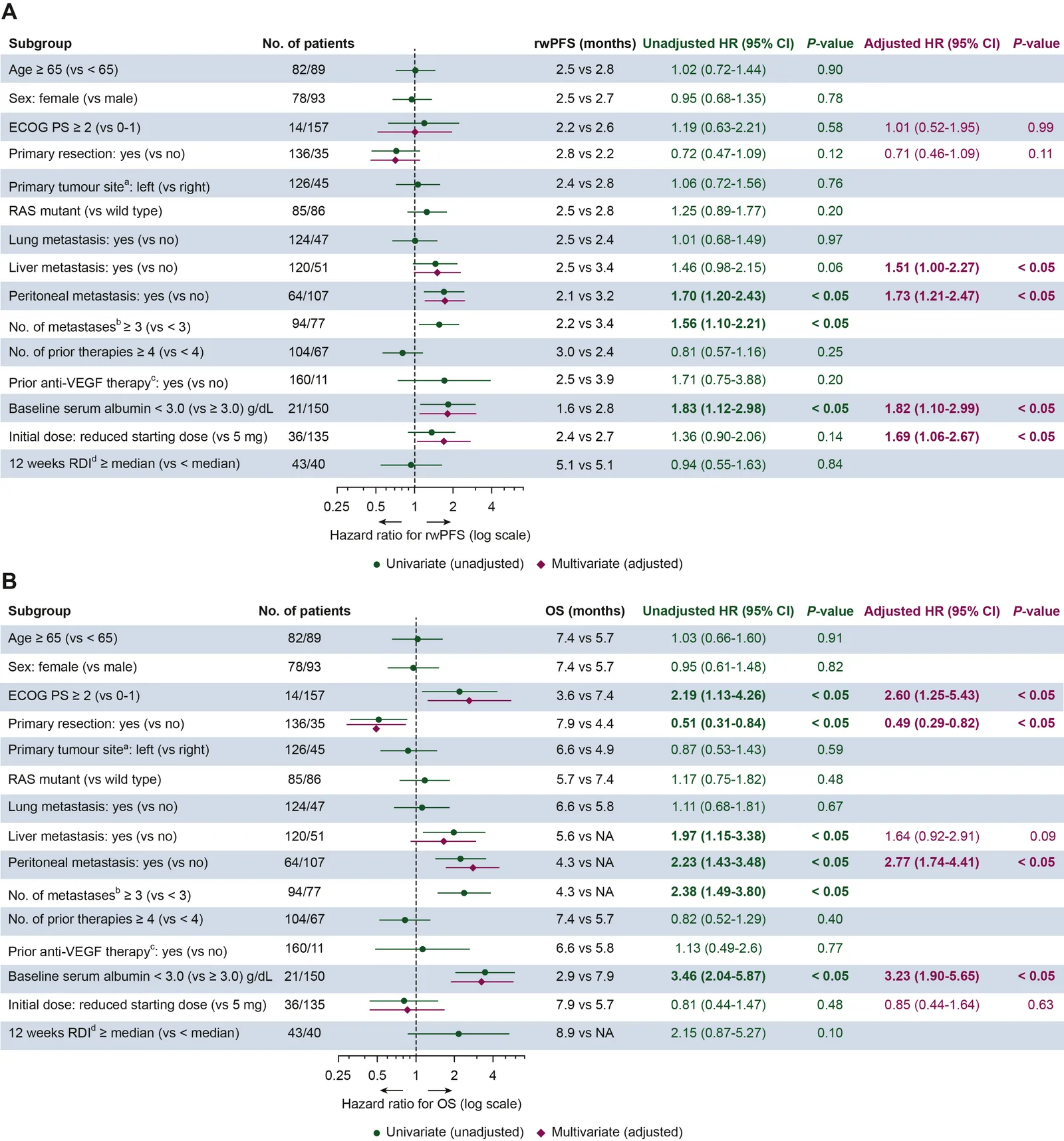
**Forest plots of HRs for (A) rwPFS and (B) OS, derived from univariate (unadjusted) and multivariate (adjusted) Cox proportional hazards models.** For each subgroup, the nonreference category is compared with its reference. Circles indicate univariate point estimates and diamonds multivariate point estimates; horizontal bars represent 95% CIs and the dashed vertical line denotes an HR of 1.0. The same set of covariates was used in the multivariate models for both endpoints. CIs, confidence intervals; ECOG PS, Eastern Cooperative Oncology Group performance status; HRs, hazard ratios; NA, not available; OS, overall survival; RDI, relative dose intensity; Ref, reference; rwPFS, real-world progression-free survival; VEGF, vascular endothelial growth factor. ^a^Right-sided tumours were from the caecum to the proximal two-thirds of transverse colon; left-sided tumours were from the distal one-third of transverse colon to the rectum. ^b^Number of metastatic sites was not included in the multivariate model to avoid potential collinearity with liver and peritoneal metastasis. ^c^Prior anti-VEGF therapy was defined as previous treatment with at least one anti-VEGF agent (bevacizumab, ramucirumab, or aflibercept). ^d^Among patients who continued fruquintinib for >12 weeks (*n* = 83).

Regarding safety, in an exploratory analysis according to the initial dose [5 mg (*n* = 135) compared with 4 or 3 mg (*n* = 36)], a reduced starting dose was not associated with a consistent reduction in toxicity (Supplementary Table S3, available at https://doi.org/10.1016/j.esmogo.2026.100418). The incidence of any-grade proteinuria was significantly higher in the reduced-dose group. Patients with baseline hypertension had a higher incidence of proteinuria, including grade ≥3 events, than those without hypertension (Supplementary Table S4, available at https://doi.org/10.1016/j.esmogo.2026.100418).

## Discussion

This multicentre, retrospective observational study evaluated the efficacy and safety of fruquintinib in patients with unresectable or recurrent CRC in Japanese clinical practice. Conducted across 23 geographically diverse institutions, this study provides real-world evidence with broad external validity. The baseline demographic, disease, and molecular characteristics of our cohort are broadly consistent with those reported in previous studies on metastatic CRC, including FRESCO-2.^3^^,^^9^^,^^10^^,^^15^, ^16^, ^17^, ^18^ In addition, the high prevalence of prior trifluridine–tipiracil plus bevacizumab exposure reflects the current treatment sequence in Japan, allowing the assessment of fruquintinib in a more contemporary clinical context than in previous pivotal studies.^9^^,^^10^^,^^19^ Nevertheless, the efficacy outcomes in our study, including rwPFS, OS, and DCR, appeared somewhat less favourable than those reported in pivotal studies. One possible explanation is that although the oncological characteristics of the study population were broadly comparable, patients in our cohort were less strictly selected with respect to treatment eligibility and baseline clinical condition. In contrast, FRESCO-2 excluded patients with ECOG PS ≥2, substantial baseline proteinuria, impaired renal function, or hepatic dysfunction. Subgroup analyses further identified both host- and tumour-related factors associated with poorer outcomes. Hypoalbuminaemia was associated with shorter survival, revealing that poor nutritional and general clinical status may influence the prognosis of patients treated with fruquintinib. In addition, liver and peritoneal metastases, multiple metastatic sites, and absence of prior primary tumour resection were associated with poorer outcomes, indicating that tumour burden and disease dissemination may influence clinical outcomes. Regarding dose-related factors, initial dose reduction was associated with shorter rwPFS in the multivariate analysis, whereas it was not an independent predictor of shorter OS after adjustment for poor prognostic factors. Because reduced starting doses were selected by treating physicians mainly for patients with poorer baseline condition and residual toxicities from prior treatment and because such indicators of frailty were only partially captured by the adjusted covariates, the association with rwPFS is likely to reflect residual confounding by indication rather than a detrimental effect of dose reduction itself. The median RDI in our cohort was lower than the 87.5% reported in the Japanese subgroup of FRESCO-2,^9^^,^^10^^,^^18^ which may partly account for the less favourable efficacy outcomes observed in our study; however, survival outcomes did not differ significantly by 12-week RDI. This should be interpreted cautiously because RDI may be influenced by treatment duration and early discontinuation. Taken together with the initial dose findings, these results reveal that individualised dose adjustments may help maintain treatment continuity in routine clinical practice.

The overall safety profile was consistent with that reported in previous studies^9^^,^^10^^,^^19^ and no new safety signals were identified. Although grade ≥3 TEAEs appeared less frequent in our cohort than in previous studies, class-related toxicities, such as hypertension, HFSR, and proteinuria, remained common, consistent with the Japanese subgroup analysis of FRESCO-2. Notably, proteinuria emerged as a clinically important toxicity in our cohort, as it was the most frequently observed AE and the leading cause of treatment modifications. This contrasts with previous studies, in which hypertension was the most frequently reported AE. This may reflect multiple factors, including the reported susceptibility of Asian patients to vascular endothelial growth factor (VEGF) inhibitor-associated proteinuria^20^ and prior exposure to agents with overlapping toxicity profiles, such as bevacizumab and regorafenib. In addition, trifluridine/tipiracil plus bevacizumab was not widely used during the FRESCO-2 study period, which may have resulted in differences in cumulative anti-VEGF exposure or anti-VEGF-free intervals. Baseline hypertension was also associated with a higher incidence of proteinuria, revealing that careful blood pressure control before and during treatment may be clinically important. These findings indicate that proteinuria management may be important for maintaining fruquintinib treatment continuity in Japanese patients.

This study has some limitations. Firstly, the retrospective design without a comparator group may have introduced a selection bias, limiting the interpretation of efficacy and safety outcomes. Secondly, lesion assessment and imaging intervals were not predefined to reflect routine clinical practice. Thirdly, confirmation of tumour response was not systematically carried out. Fourthly, the 12-week RDI analysis was restricted to patients who remained on treatment for at least 12 weeks, potentially introducing survivor bias and limiting its interpretation. Finally, the observation period was relatively short. These limitations are inherent to retrospective real-world analyses and prospective validation is warranted; the results of the ongoing FruBLOOM trial (JACCRO CC-19)^21^ are awaited.

### Conclusion

Fruquintinib demonstrated modest real-world activity in Japanese patients treated in routine clinical practice, with no new safety signals identified. However, proteinuria occurs frequently and represents a major cause of treatment modification. These findings indicate that, along with antitumour activity, careful toxicity management and sustained treatment continuity are crucial for maximising the clinical benefits of fruquintinib in this setting. Further prospective studies are required to establish optimal dosing and dose modification strategies.
