# Supplementary material for: Real-world efficacy, safety, and treatment continuity of fruquintinib in Japanese patients with colorectal cancer: a multicentre retrospective study
Source: ESMO Gastrointest Oncol. 2026 Sep 22;14:100418. doi: 10.1016/j.esmogo.2026.100418 (PMC13633917; doi:10.1016/j.esmogo.2026.100418)
Supplement: Supplementary file 1 [file mmc1.docx]

**Supplementary Data**

**Supplementary Table 1.** Univariate and multivariate subgroup analyses of rwPFS

|  | n | rwPFS | | | | |
| --- | --- | --- | --- | --- | --- | --- |
|  |  | Median, months (95% CI) | ***Univariate analysis*** | | ***Multivariate analysis*** | |
|  |  |  | HR (95% CI) | *p*-value | HR (95% CI) | *p*-value |
| Age, years  < 65  ≥ 65 | 89  82 | 2.8 (2.2–3.4)  2.5 (2.2–3.6) | Ref  1.02 (0.72–1.44) | 0.90 |  |  |
| Sex  Male  Female | 93  78 | 2.7 (2.2–3.6)  2.5 (2.1–3.4) | Ref  0.95 (0.68–1.35) | 0.78 |  |  |
| ECOG PS  0–1  ≥ 2 | 157  14 | 2.6 (2.3–3.2)  2.2 (1.4–3.7) | Ref  1.19 (0.63–2.21) | 0.58 | Ref  1.01 (0.52–1.95) | 0.99 |
| Primary resection  No  Yes | 35  136 | 2.2 (1.8–2.8)  2.8 (2.4–3.7) | Ref  0.72 (0.47–1.09) | 0.12 | Ref  0.71 (0.46–1.09) | 0.11 |
| Primary tumour site  Right  Left | 45  126 | 2.8 (2.3–3.8)  2.4 (2.2–3.2) | Ref  1.06 (0.72–1.56) | 0.76 |  |  |
| RAS  Wild type  Mutant | 86  85 | 2.8 (2.3–3.8)  2.5 (2.0–3.0) | Ref  1.25 (0.89–1.77) | 0.20 |  |  |
| Lung metastasis  No  Yes | 47  124 | 2.4 (2.0–3.9)  2.5 (2.3–3.2) | Ref  1.01 (0.68–1.49) | 0.97 |  |  |
| Liver metastasis  No  Yes | 51  120 | 3.4 (2.2–4.8)  2.5 (2.2–3.0) | Ref  1.46 (0.98–2.15) | 0.06 | Ref  1.51 (1.00–2.27) | < 0.05 |
| Peritoneal metastasis  No  Yes | 107  64 | 3.2 (2.5–3.8)  2.1 (1.6–2.3) | Ref  1.70 (1.20–2.43) | < 0.05 | Ref  1.73 (1.21–2.47) | < 0.05 |
| Number of metastases  < 3  ≥ 3 | 77  94 | 3.4 (2.7–3.9)  2.2 (1.8–2.5) | Ref  1.56 (1.10–2.21) | < 0.05 |  |  |
| Number of prior therapies  < 4  ≥ 4 | 67  104 | 2.4 (1.9–2.8)  3.0 (2.3–3.8) | Ref  0.81 (0.57–1.16) | 0.25 |  |  |
| Prior anti-VEGF therapy  No  Yes | 11  160 | 3.9 (2.3–NA)  2.5 (2.2–3.0) | Ref  1.71 (0.75–3.88) | 0.20 |  |  |
| Baseline serum albumin  ≥ 3.0 g/dL  < 3.0 g/dL | 150  21 | 2.8 (2.4–3.6)  1.6 (0.7–2.5) | Ref  1.83 (1.12–2.98) | < 0.05 | Ref  1.82 (1.10–2.99) | < 0.05 |
| Initial dose  5 mg  reduced starting dose | 135  36 | 2.7 (2.3–3.4)  2.4 (1.6–3.6) | Ref  1.36 (0.90–2.06) | 0.14 | Ref  1.69 (1.06–2.67) | < 0.05 |
| 12 weeks RDI^a^  < Median  ≥ Median | 40  43 | 5.1 (3.7–6.7)  5.1 (3.9–6.2) | Ref  0.94 (0.55–1.63) | 0.84 |  |  |

^a^Among patients who continued fruquintinib over 12 weeks (n = 83)

Abbreviations: rwPFS, real-world progression-free survival; HR, hazard ratio; Ref, reference; ECOG PS, Eastern Cooperative Oncology Group performance status; VEGF, vascular endothelial growth factor; RDI, relative dose intensity.

**Supplementary Table 2.** Univariate and multivariate subgroup analyses of OS

|  | **n** | **OS** | | | | |
| --- | --- | --- | --- | --- | --- | --- |
|  |  | **Median, months (95% CI)** | ***Univariate analysis*** | | ***Multivariate analysis*** | |
|  |  |  | **HR (95% CI)** | ***p*-value** | **HR (95% CI)** | ***p*-value** |
| Age, years  < 65  ≥ 65 | 89  82 | 5.7 (4.4–NA)  7.4 (4.7–NA) | Ref  1.03 (0.66–1.60) | 0.91 |  |  |
| Sex  Male  Female | 93  78 | 5.7 (4.7–NA)  7.4 (4.4–NA) | Ref  0.95 (0.61–1.48) | 0.82 |  |  |
| ECOG PS  0–1  ≥ 2 | 157  14 | 7.4 (5.6–NA)  3.6 (2.3–4.9) | Ref  2.19 (1.13–4.26) | < 0.05 | Ref  2.60 (1.25–5.43) | < 0.05 |
| Primary resection  No  Yes | 35  136 | 4.4 (2.8–5.7)  7.9 (5.7–NA) | Ref  0.51 (0.31–0.84) | < 0.05 | Ref  0.49 (0.29–0.82) | < 0.05 |
| Primary tumour site^a^  Right  Left | 45  126 | 4.9 (3.7–NA)  6.6 (5.6–NA) | Ref  0.87 (0.53–1.43) | 0.59 |  |  |
| RAS  Wild type  Mutant | 86  85 | 7.4 (4.9–NA)  5.7 (4.3–NA) | Ref  1.17 (0.75–1.82) | 0.48 |  |  |
| Lung metastasis  No  Yes | 47  124 | 5.8 (4.9–NA)  6.6 (4.4–NA) | Ref  1.11 (0.68–1.81) | 0.67 |  |  |
| Liver metastasis  No  Yes | 51  120 | NA (5.6–NA)  5.6 (4.2–7.4) | Ref  1.97 (1.15–3.38) | < 0.05 | Ref  1.64 (0.92–2.91) | 0.09 |
| Peritoneal metastasis  No  Yes | 107  64 | NA (5.7–NA)  4.3 (3.2–6.6) | Ref  2.23 (1.43–3.48) | < 0.05 | Ref  2.77 (1.74–4.41) | < 0.05 |
| Number of metastases^b^  < 3  ≥ 3 | 77  94 | NA (5.7–NA)  4.3 (3.7–7.4) | Ref  2.38 (1.49–3.80) | < 0.05 |  |  |
| Number of prior therapies  < 4  ≥ 4 | 67  104 | 5.7 (4.3–NA)  7.4 (4.9–NA) | Ref  0.82 (0.52–1.29) | 0.40 |  |  |
| Previous anti-VEGF therapy^c^  No  Yes | 11  160 | 5.8 (3.3–NA)  6.6 (4.8–8.9) | Ref  1.13 (0.49–2.6) | 0.77 |  |  |
| Baseline serum albumin  ≥ 3.0 g/dL  < 3.0 g/dL | 150  21 | 7.9 (5.7–NA)  2.9 (1.9–3.4) | Ref  3.46 (2.04–5.87) | < 0.05 | Ref  3.23 (1.90–5.65) | < 0.05 |
| Initial dose  5 mg  Reduced starting dose | 135  36 | 5.7 (4.7–8.9)  7.9 (4.9–NA) | Ref  0.81 (0.44–1.47) | 0.48 | Ref  0.85 (0.44–1.64) | 0.63 |
| 12 weeks RDI^d^  < Median  ≥ Median | 40  43 | NA (NA–NA)  8.9 (5.7–NA) | Ref  2.15 (0.87–5.27) | 0.10 |  |  |

^a^ Right-sided included tumours from the caecum to the proximal two-thirds of transverse colon; left-sided represented tumours from the distal one-third of transverse colon to the rectum.

^b^　Number of metastatic sites was not included in the multivariate model to avoid potential collinearity with liver and peritoneal metastasis.

^c^ Prior anti-VEGF therapy was defined as previous treatment with at least one anti-VEGF agent (bevacizumab, ramucirumab, or aflibercept).

^d^ Among patients who continued fruquintinib over 12 weeks (n = 83)

Abbreviations: OS, overall survival; HR, hazard ratio; Ref, reference; ECOG PS, Eastern Cooperative Oncology Group performance status; VEGF, vascular endothelial growth factor; RDI, relative dose intensity; NA, not available

**Supplementary Table 3.** Comparison of treatment-emergent adverse events according to initial dose

|  | | **5 mg** | **Reduced starting dose^a^** |  |
| --- | --- | --- | --- | --- |
|  |  | **n = 135** | **n = 36** | ***p*-value** |
| All TEAEs | Any grade | 130 (96.3) | 34 (94.4) | 0.64 |
|  | Grade ≥ 3 | 67 (49.6) | 17 (47.2) | 0.85 |
| Proteinuria | Any grade | 66 (48.9) | 28 (77.8) | < 0.05 |
|  | Grade ≥ 3 | 30 (22.2) | 8 (22.2) | 1.00 |
| Fatigue | Any grade | 68 (50.4) | 15 (41.7) | 0.45 |
|  | Grade ≥ 3 | 6 (4.4) | 0 (0.0) | 0.35 |
| HFSR | Any grade | 56 (41.5) | 18 (50.0) | 0.45 |
|  | Grade ≥ 3 | 5 (3.7) | 2 (5.6) | 0.64 |
| Treatment emergent  Hypertension | Any grade | 53 (39.3) | 20 (55.6) | 0.09 |
|  | Grade ≥ 3 | 19 (14.1) | 7 (19.4) | 0.44 |
| Anorexia | Any grade | 47 (34.8) | 7 (19.4) | 0.11 |
|  | Grade ≥ 3 | 6 (4.4) | 0 (0.0) | 0.35 |
| AST increased | Any grade | 42 (31.1) | 9 (25.0) | 0.54 |
|  | Grade ≥ 3 | 7 (5.2) | 1 (2.8) | 1.00 |
| Platelet count decreased | Any grade | 36 (26.7) | 8 (22.2) | 0.67 |
|  | Grade ≥ 3 | 4 (3.0) | 1 (2.8) | 1.00 |
| ALT increased | Any grade | 29 (21.5) | 5 (13.9) | 0.36 |
|  | Grade ≥ 3 | 3 (2.2) | 1 (2.8) | 1.00 |
| Anaemia | Any grade | 31 (23.0) | 3 (8.3) | 0.06 |
|  | Grade ≥ 3 | 3 (2.2) | 0 (0.0) | 1.00 |
| Nausea | Any grade | 25 (18.5) | 1 (2.8) | < 0.05 |
|  | Grade ≥ 3 | 2 (1.5) | 0 (0.0) | 1.00 |
| Diarrhoea | Any grade | 22 (16.3) | 2 (5.6) | 0.11 |
|  | Grade ≥ 3 | 3 (2.2) | 0 (0.0) | 1.00 |
| Mucositis oral | Any grade | 20 (14.8) | 1 (2.8) | 0.08 |
|  | Grade ≥ 3 | 1 (0.7) | 0 (0.0) | 1.00 |
| T-Bil increased | Any grade | 18 (13.3) | 1 (2.8) | 0.08 |
|  | Grade ≥ 3 | 6 (4.4) | 0 (0.0) | 0.35 |

^a^Reduced dose included 3 or 4 mg

**Supplementary Table 4.** Comparison of treatment-emergent adverse events according to baseline hypertension

|  | | **Baseline**  **hypertension** | **Baseline**  **non-hypertension** |  |
| --- | --- | --- | --- | --- |
|  |  | **n = 88** | **n = 83** | ***p*-value** |
| All TEAEs | Any grade | 87 (98.9) | 77 (92.8) | 0.06 |
|  | Grade ≥ 3 | 45 (51.1) | 39 (47.0) | 0.65 |
| Proteinuria | Any grade | 61 (69.3) | 33 (39.8) | < 0.05 |
|  | Grade ≥ 3 | 26 (29.5) | 12 (14.5) | < 0.05 |
| Fatigue | Any grade | 40 (45.5) | 43 (51.8) | 0.45 |
|  | Grade ≥ 3 | 1 (1.1) | 5 (6.0) | 0.11 |
| HFSR | Any grade | 38 (43.2) | 36 (43.4) | 1.00 |
|  | Grade ≥ 3 | 3 (3.4) | 4 (4.8) | 0.71 |
| Treatment-emergent　hypertension | Any grade | 45 (51.1) | 28 (33.7) | < 0.05 |
|  | Grade ≥ 3 | 17 (19.3) | 9 (10.8) | 0.14 |
| Anorexia | Any grade | 28 (31.8) | 26 (31.3) | 1.00 |
|  | Grade ≥ 3 | 2 (2.3) | 4 (4.8) | 0.43 |
| AST increased | Any grade | 30 (34.1) | 21 (25.3) | 0.24 |
|  | Grade ≥ 3 | 3 (3.4) | 5 (6.0) | 0.49 |
| Platelet count decreased | Any grade | 22 (25.0) | 22 (26.5) | 0.86 |
|  | Grade ≥ 3 | 2 (2.3) | 3 (3.6) | 0.68 |
| ALT increased | Any grade | 19 (21.6) | 15 (18.1) | 0.70 |
|  | Grade ≥ 3 | 2 (2.3) | 2 (2.4) | 1.00 |
| Anaemia | Any grade | 17 (19.3) | 17 (20.5) | 0.85 |
|  | Grade ≥ 3 | 2 (2.3) | 1 (1.2) | 1.00 |
| Nausea | Any grade | 12 (13.6) | 14 (16.9) | 0.67 |
|  | Grade ≥ 3 | 1 (1.1) | 1 (1.2) | 1.00 |
| Diarrhoea | Any grade | 9 (10.2) | 15 (18.1) | 0.19 |
|  | Grade ≥ 3 | 0 (0.0) | 3 (3.6) | 0.11 |
| Mucositis oral | Any grade | 7 (8.0) | 14 (16.9) | 0.10 |
|  | Grade ≥ 3 | 0 (0.0) | 1 (1.2) | 0.49 |
| Total bilirubin increased | Any grade | 8 (9.1) | 11 (13.3) | 0.47 |
|  | Grade ≥ 3 | 3 (3.4) | 3 (3.6) | 1.00 |

Abbreviations: TEAEs, treatment-emergent adverse events; HFSR, hand–foot skin reaction; AST, aspartate aminotransferase; ALT, alanine aminotransferase
